# The imaging dynamic changes in the malignant transformation of an epidermoid cyst: a case report and literature review

**DOI:** 10.3389/fneur.2024.1349044

**Published:** 2024-02-14

**Authors:** Tian Yang, Jibo Hu, Lele Li, Houyun Xu, Caijuan Zhang, Zhilan Huang, Jun Yang, Huiqing Zhang

**Affiliations:** ^1^Department of Radiology, The Fourth Affiliated Hospital of School of Medicine, and International School of Medicine, International Institutes of Medicine, Zhejiang University, Yiwu, China; ^2^Department of Clinical Laboratory, The Fourth Affiliated Hospital of School of Medicine, and International School of Medicine, International Institutes of Medicine, Zhejiang University, Yiwu, China

**Keywords:** epidermoid cysts, malignant transformation, squamous cell carcinoma, pre-pontine cistern, follow-up

## Abstract

Malignant transformation of epidermoid cysts is a rare complication. Most of the previously reported cases have involved postoperative malignant transformations. We present a case of malignant transformation of a nonpostoperative epidermoid tumor into squamous cell carcinoma (SCC) that occurred in a 61-year-old Chinese woman. The patient’s initial cranial MRI scan showed an epidermoid cyst with marginal enhancement in the pre-pontine cistern, and the lesion gradually enlarged after 10 months. A craniotomy was performed using to remove part of the tumor via the right retrosigmoid approach, and postoperative pathology confirmed that the transformation of the epidermoid cyst was malignant. Our case study suggests that the possibility of malignant transformation of epidermoid cyst should not be ignored on the basis of enhanced imaging features, regardless of whether they are nodular, annular, or patchy, as is the case for inflammation. Strict follow-up is required for early detection of malignant transformation to prompt correspondingly early clinical treatment.

## Introduction

An intracranial epidermoid cyst is a rare developmental benign tumor whose malignant transformation is very rare and has been poorly reported. Most of the reported cases involved postoperative malignant transformation, and cases without surgical treatment for malignant transformation are even rarer. In this paper, we report a case in which malignant transformation of an epidermoid cyst was confirmed by pathology and describe its dynamic imaging manifestations within 2 years to improve the clinical understanding of the malignant transformation of a cranial epidermoid cyst.

## Case report

**Figure 1:**
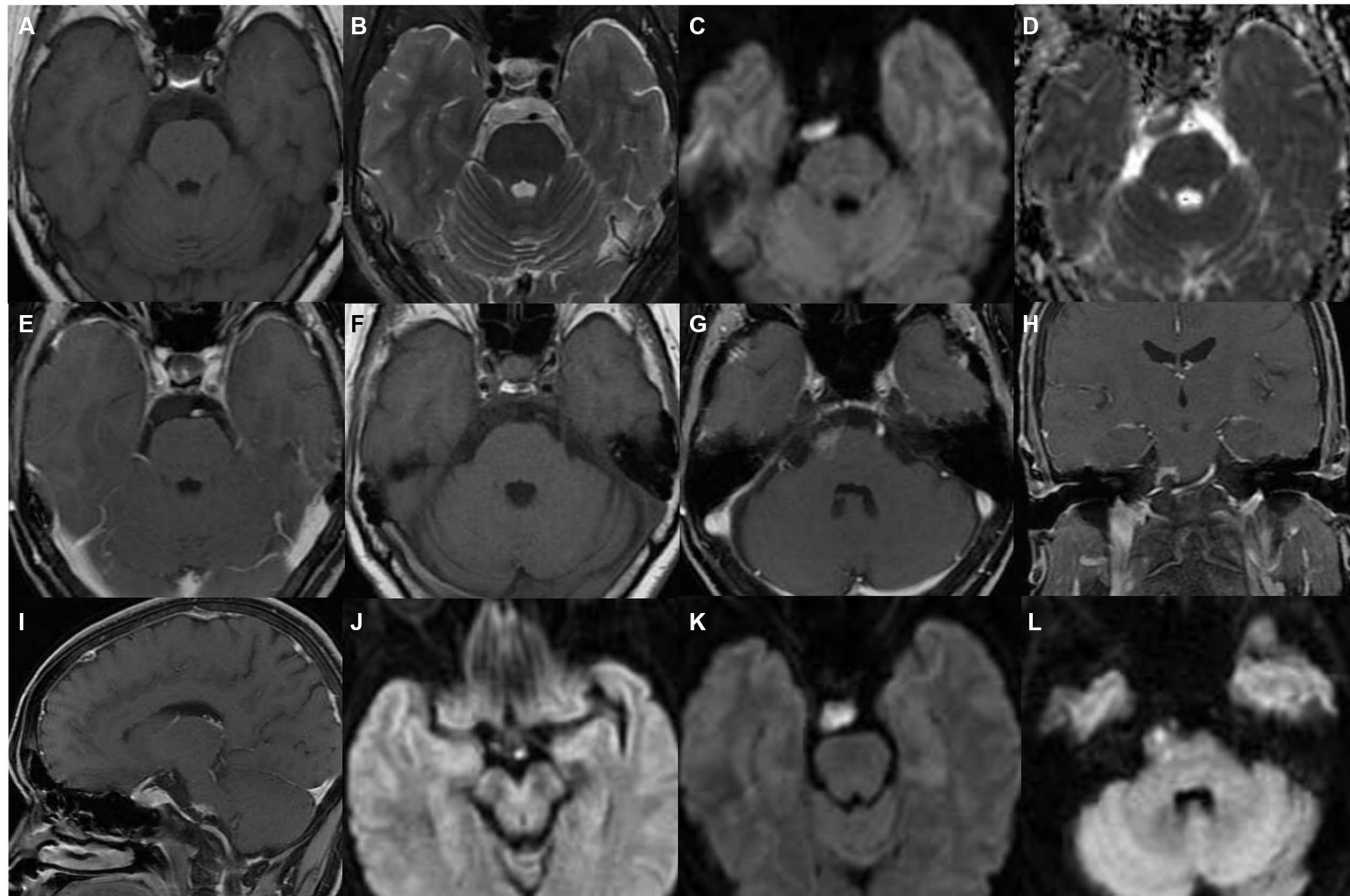
MRI in December 2021. **(A−E)** The nodule in the pre-pontine cistern showed a low-intensity signal on T1-weighted imaging, a high-intensity signal on T2-weighted imaging, a high-intensity signal on diffusion-weighted imaging (DWI), a low-intensity signal on an apparent diffusion coefficient plot (ADC), and no enhancement on contrast-enhanced T1-weighted imaging; **(F–I)** the lesion is adjacent to the right edge of the pons and shows patch-like enhancement, the boundary is not clear; **(C,J–L)** the lesion extends from the optic chiasm to the beginning of the right abducens nerve, the lesion is in close proximity to the right trigeminal nerve, abducens nerve, and optic chiasm nerve.

A 61-year-old female patient was admitted to the Department of Neurosurgery, the Fourth Affiliated Hospital of Zhejiang University School of Medicine, due to restricted right eye abduction. The patient had progressive worsening of symptoms for 8 months, accompanied by blurred vision and double vision. She had no history of tumors or brain disorders, and no family members had any related medical history. Laboratory tests did not reveal abnormalities, such as tumor markers or thrombotic markers. On December 28, 2021, a cranial MRI scan revealed a nodular abnormal signal in the pre-pontine cistern with unclear borders and irregular morphology, which was consistent with the imaging findings of epidermoid cysts (Figures 1A−E). However, a patchy enhancement lesion was shown in the adjacent pons, and the clinician thought that this feature might due to inflammation in the pons adjacent to the epidermoid cyst and could not rule out the possibility of a tumor (Figures 1F−I). The lesion extends from the optic chiasm to the beginning of the right abducens nerve, the lesion is in close proximity to the right trigeminal nerve, abducens nerve, and optic chiasm nerve (Figures 1C,J–L). The patient and their families refused surgery because of the high risk of surgery. Doctors recommended treatment with methylprednisolone sodium succinate to improve symptoms of abducens nerve irritation and instructed the patient regarding the need for close follow-up. However, the patient did not pay enough attention to the treatment and stopped treatment independently after 8 days.

**Figure 2:**
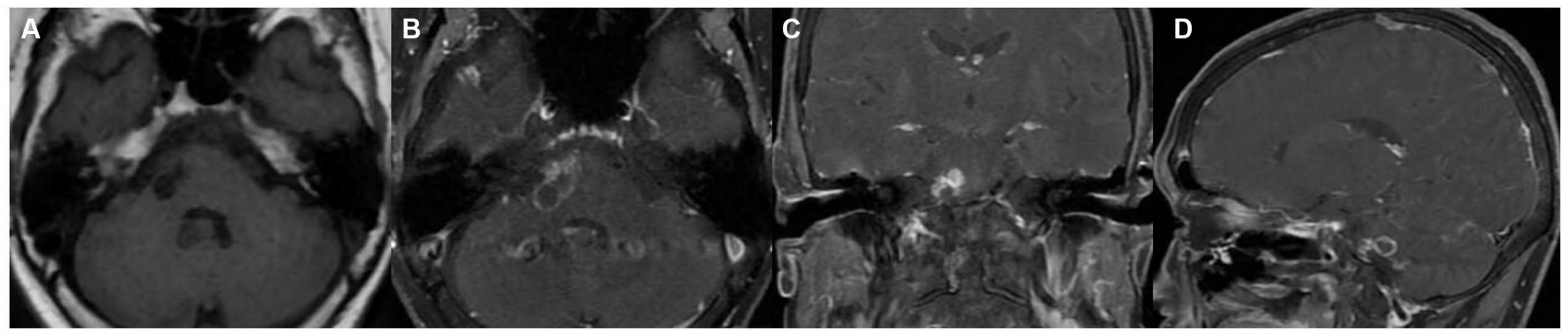
Imaging taken in July 2022. **(A)** Cystic lesions are visible on the right side of the pons on T1-weighted imaging; **(B−D)** contrast-enhanced scan image showing that the lesion had a ring shape and nodular enhancement features, and the lesion invaded the brachium pontis and was considered to be a neoplastic lesion.

**Figure 3:**
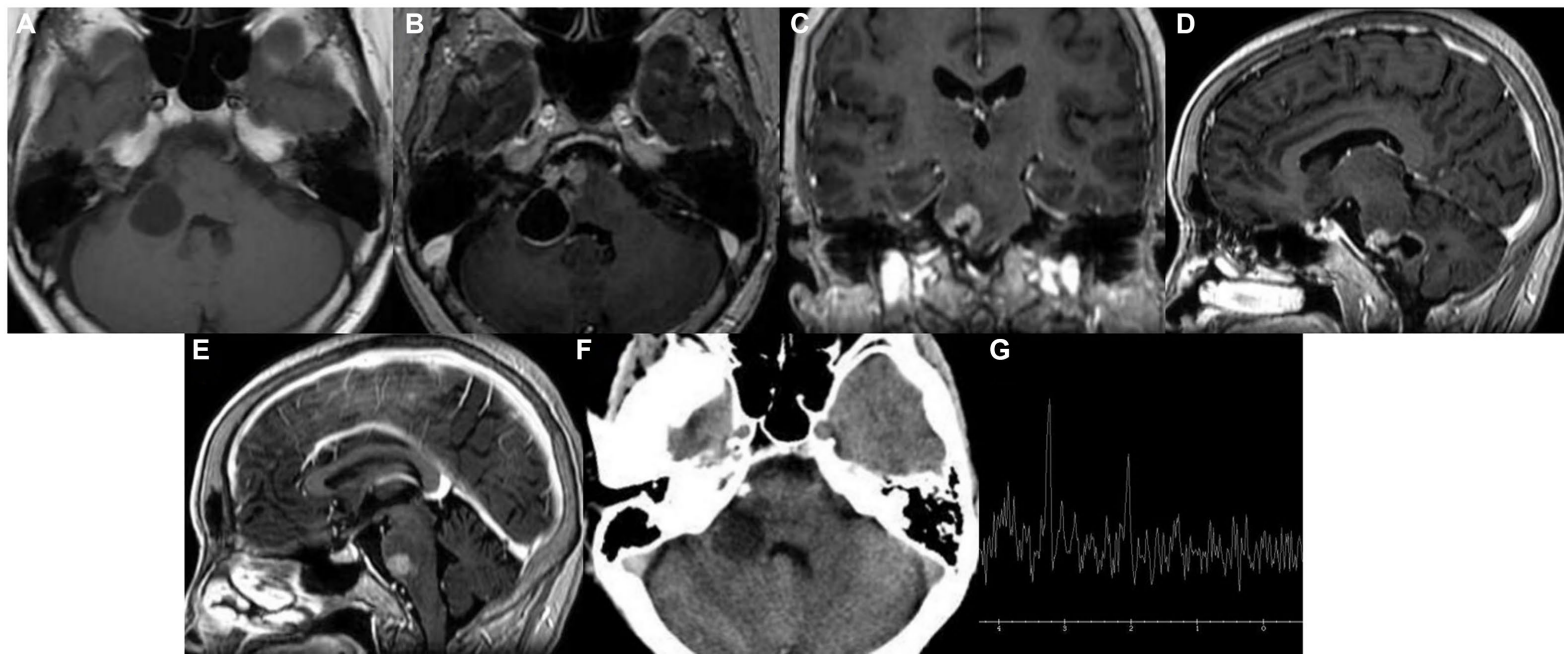
Imaging examination in October 2022. **(A−D)** A cystic solid shadow was observed in the right pons, brachium pontis, and right cerebellar hemisphere; **(E)** the adjacent meninges were slightly thickened; **(F)** speckled calcification appeared in the low-density lesion area; and **(G)** single-voxel 1H + MR spectroscopy (MRS).

On July 6, 2022, the patient underwent another MRI scan, which showed that the right pontine edge enhancement lesion was larger than it was on December 28, 2021, and that cystic lesions had appeared (Figure 2). The lesion at the right margin of the pons exhibited annular enhancement and extended into the brachium pontis. On October 10, 2022, the patient underwent another cranial computerized tomography (CT), a magnetic resonance enhancement scan, and magnetic resonance spectroscopy (MRS) due to worsening symptoms. The results showed that the lesion was further expanded than it was on July 6, 2022 (Figures 3A−D). A cystic solid lesion was revealed in the immediate vicinity of the right pontine border, the right brachium pontis, and the right cerebellar hemisphere. The boundaries of the cystic-solid lesions were unclear, and the solid component was markedly enhanced. The lesions were in close proximity to the right abducens nerve, some lesions invaded the brainstem, and the meninges adjacent to the brainstem were thickened (Figure 3E). CT of the brain showed an area of low-density lesions with speckled calcifications (Figure 3F). The area of the lesion located in the pontine and right brachium pontis showed an elevated Cho peak, a decreased NAA peak, and a Lac peak (Figure 3G). At the same time, the patient underwent a comprehensive examination, and no obvious tumor signs were found outside the brain.

**Figure 4:**
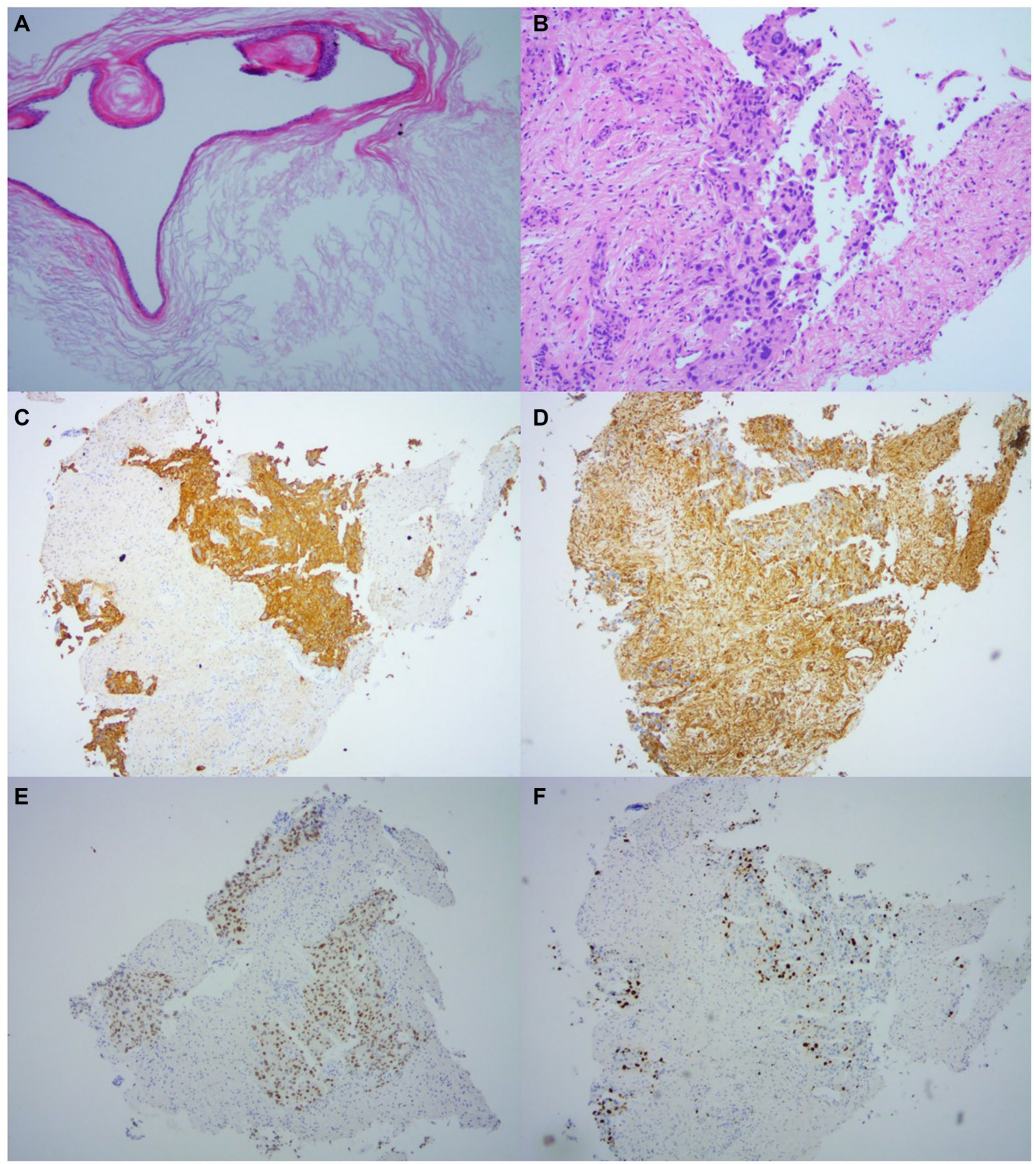
Histopathology. **(A)** Histopathology taken from the anterior pre-pontine cistern shows lamellar keratoses with a stratified squamous epithelium lined with a sac wall, consistent with the characteristics of an epidermoid cyst (HE × 100). **(B)** Histopathology taken from within the brainstem shows carcinoma invasion (HE × 100). **(C–F)** IHC results were taken from brainstem lesions: CK (AE1/AE3) was positive **(C)**, GATA3 **(D)** was positive, Vimentin was focal-expressing **(E)**, and Ki-67 proliferation index was 20%.

On October 13, 2022, the doctor performed a craniotomy to remove part of the tumor via the right retrosigmoid approach. Histopathological examination of tissue taken from the anterior pre-pontine cistern showed features consistent with epidermoid cysts (Figure 4A). Histopathological results of tissue taken from the brainstem showed invasion by carcinoma (Figure 4B); immunohistochemistry (IHC) confirmed that CK (AE1/AE3) was positive (Figure 4C), GATA3 (Figure 4D) was positive, vimentin was focally expressed (Figure 4E), GFAP, S-100, IDH-1, Oligo-2, TTF-1, PAX8, CK20, and CDX-2 were negative, and the Ki-67 proliferation index was 20% (Figure 4F).

**Figure 5:**
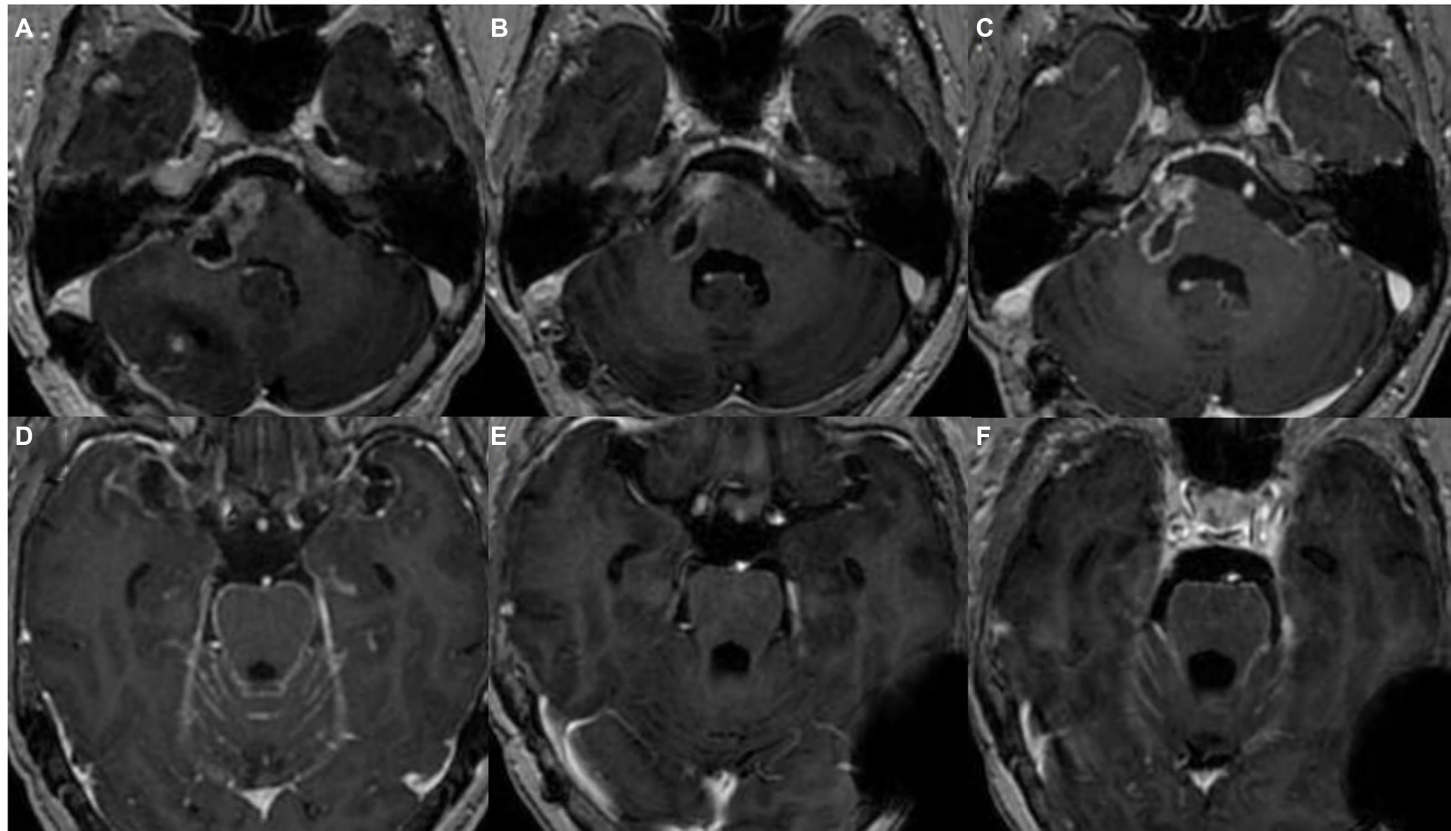
MRI-enhanced image of postoperative lesions. **(A)** The image of some unresected lesions in the brainstem area before postoperative radiotherapy; **(B)** unresected cystic solid lesions in the brainstem on January 6, 2023; **(C)** the image of brainstem lesions on February 25, 2023; **(D)** diffuse meningeal metastases on February 25, 2023; **(E)** diffuse meningeal metastases on July 6, 2023; and **(F)** diffuse meningeal metastases on October 06, 2023.

On October 18, 2022, the patient underwent the first cranial MRI enhancement examination (Figure 5A) after surgery, and the patient underwent radiation therapy in another hospital for 1 month. On January 6, 2023 (Figure 5B), magnetic resonance enhancement revealed that the unresected cystic solid lesions in the brainstem were slightly smaller than on October 18, 2022. On February 25, 2023, the lesions (Figure 5C) were slightly larger than before, with diffuse meningeal metastases (Figure 5D). Ventriculoperitoneal shunt was performed on April 6, 2023, and six cycles of bevacizumab (avastin) combined with nab-paclitaxel combined with carboplatin chemotherapy were performed from April 24, 2023 to September 22, 2023. During this period, on July 6, 2023 (Figure 5E), MRI showed that the condition of cranial metastases has improved compared to before. On October 06, 2023, the cranial metastases (Figure 5F) progressed than it was on July 6, 2023, and chemotherapy was performed with temozolomide. The patient was hospitalized on October 31, 2023 due to terminal tumor and lung infection, and unfortunately passed away on November 11, 2023 due to worsening of his condition.

## Discussion

During normal embryonic development, there is no epithelial tissue in the human brain. Therefore, the vast majority of intracranial squamous cell carcinomas (SCCs) are secondary to distant metastasis of primary malignancies, such as head and neck cancer and lung cancer. Since 1912, when Ens first proposed the malignant transformation of epidermoid cysts, corresponding studies have been performed, most of which involved postoperative malignant transformations, which occurred only rarely. Cases of malignant transformation without surgical treatment, as reported here, are even rarer.

At present, the reported malignant transformation of epidermoid cysts mostly occurs in middle-aged female patients, with an average age of 54 years (1–3). The most common location is the cerebellopontine angle, accounting for approximately 61.5% of cases. The average postoperative malignant transformation time is 82.7 months, and the prognosis is poor (1). It has been reported that patients who have undergone surgery alone have survived the shortest time to die within 1 month, and it is very rare to survive for more than 5 years (4). The lesion in a 61-year-old woman described in this article originated from the pre-pontine cistern, developed diffuse meningeal metastases only 4 months after surgery, and unfortunately led to the patient passing away 13 months after surgery. However, the mechanism of malignant transformation in epidermoid cysts is unclear, and studies have suggested that chronic inflammation caused by epidermoid cyst rupture or surgical foreign body presence and incomplete surgical resection may be the main factors involved (5). Several studies have suggested that when squamous metaplasia or *p53* mutation is observed in pathological specimens of epidermoid cysts, vigilance is needed for malignant transformation (1). This novel study reported the malignant transformation of an epidermoid cyst in the pineal region in which gene sequencing revealed a low mutational burden (l Mut/Mb), microsatellite stability, and loss of *CDKN* 2A/B, *MTAP* (exons 2–8), and *PTEN* (exons 6–9) (6). IHC of malignant transformation of epidermoid cysts is positive for CK and EMA (7–9). In this case, IHC revealed CK and GATA3 expression, which was similar to what was previously reported.

Epidermoid cysts are slow-growing, benign lesions that are usually asymptomatic for many years. Epidermoid cysts are variable and exhibit multiple outcomes as the disease progresses, such as resolution, hemorrhage, and malignant transformation (10–12); however, when symptoms and signs develop rapidly, enhancement of lesion margins and nodular enhancement suggest the possibility of malignant transformation of epidermoid cysts (13). Patchy enhancement of adjacent tissues of epidermoid cysts has been reported to indicate chemical meningitis, but malignant transformation should not be ruled out (14). To rule out malignant transformation, it is necessary to observe the patient’s signs, morphology, and progression of the lesion, such as whether there is prominent rapid tumor growth, tissue edema, and nodular enhancement in adjacent or cystic lesions. The initial imaging results of the patient we reported showed patchy enhancement of the lesion adjacent to the pons, and the initial diagnosis was chemical inflammation around the epidermoid cyst. After 7 months of follow-up, the patient’s symptoms progressively worsened, the lesion was obviously enlarged with necrosis, the adjacent brain tissue was invaded, and edema bands had formed around the lesion. MRS showed an increase in the Cho peak. The nonsignificant decrease in NAA peak intensity may have been due to the inclusion of normal tissue at the time of selection of the region of interest. Nodular leptomeningeal enhancement is an early imaging sign of leptomeningeal metastasis after malignant transformation of epidermoid cysts (14). Similarly, the last MRI of this patient before surgery showed lesion invasion of the brainstem and thickening of the meninges near the brainstem, which indicated leptomeningeal invasion. Diffuse meningeal metastases developed 4 months postoperatively and subsequently progressively worsened.

Imaging findings of epidermoid cyst malignancy are not specific and often present with enhancement features of the lesion, with or without cerebral edema. It has been suggested (15) that enhancement of epidermoid cyst margins or nodular enhancement is usually a sign of malignant transformation, and patchy enhancement of lesion margins is often thought to be chemical meningitis due to cyst rupture, but atypical for malignant transformation. It has also been suggested (10) that almost all cases have worsening of signs and different degrees of enhancement of lesions before malignant transformation, so atypical enhancement methods need to be combined with patients’ signs to make the best diagnosis.

The most important differentiating factor of intracranial epidermoid cyst malignancy on imaging is metastasis; therefore, when a single intracranial lesion is present, a comprehensive examination should also be performed to rule out the diagnosis of extracranial metastases. In this case, after comprehensive inspection to rule out metastatic tumors, malignant transformation of the epidermoid cyst was diagnosed according to the pathological results, which were consistent with the criteria of Hamlat et al. (16). The history of early disease progression was unknown because the patient did not undergo relevant tests before starting to experience symptoms or within 8 months of symptom onset. This case is the first cases diagnosed in our hospital; moreover, there are no other case data for comparison, and it is still necessary to summarize the characteristics of similar cases.

## Conclusion

Although epidermoid cysts are mostly benign and malignant transformation is rare, it does occur. Patients with epidermoid cysts should be followed up, either surgically or conservatively. In addition, if there are nodal, annular or patchy imaging enhancement features, such as is in inflammation, the possibility of malignant transformation of epidermoid cysts cannot be ignored, and close follow-up is required for early detection of malignant transformation to prompt correspondingly early clinical treatment.

## Data availability statement

The original contributions presented in the study are included in the article/supplementary material; further inquiries can be directed to the corresponding authors.

## Ethics statement

The studies involving humans were approved by Human Research Ethics Committee of the Fourth Affiliated Hospital of Zhejiang University School of Medicine. The studies were conducted in accordance with the local legislation and institutional requirements. Written informed consent for participation was not required from the participants or the participants’ legal guardians/next of kin in accordance with the national legislation and institutional requirements. Written informed consent was obtained from the individual(s) for the publication of any potentially identifiable images or data included in this article.

## Author contributions

TY: Funding acquisition, Writing – original draft, Writing – review & editing. JH: Writing – original draft, Writing – review & editing. LL: Funding acquisition, Writing – review & editing. HX: Writing – review & editing. CZ: Writing – review & editing. ZH: Writing – review & editing. JY: Funding acquisition, Resources, Writing – review & editing. HZ: Resources, Writing – review & editing.
